# MRI-confirmed cochlear artery infarct clinically diagnosed in a patient with sickle cell disease: a case report

**DOI:** 10.1007/s00405-024-08837-0

**Published:** 2024-07-18

**Authors:** Michaël Risoud, Philippine Toulemonde, Cyril Beck, Quentin Charley, Édouard Suzzoni, Christophe Vincent, Frédérique Dubrulle

**Affiliations:** 1 Department of Otology and Neurotology, CHU Lille, Lille, F-59000 France; 2 Medical Imagin Center, CHU Lille, Lille, F-59000 France; 3 Department of Otology and Neurotology, Hôpital Roger Salengro, University Hospital of Lille (CHU Lille), rue Emile Laine, Lille cedex, 59037 France

**Keywords:** MRI, 3D FLAIR HR, Cochlear artery infarct, Sickle cell disease, Sudden sensorineural hearing loss

## Abstract

**Purpose:**

To corroborate the vascular etiology of sudden sensorineural hearing loss (SNHL) utilizing magnetic resonance imaging (MRI).

**Patient:**

A 24-year-old male with a history of sickle cell disease experienced sudden SNHL and right horizontal nystagmus, without accompanying vertigo.

**Intervention:**

Audiometric evaluation revealed left-sided SNHL, predominantly affecting high frequencies. Video head impulse testing demonstrated isolated dysfunction of the left posterior semicircular canal. An urgent brain MRI identified a recent punctiform ischemic stroke in the frontal region. A subsequent MRI, conducted with a 4-hour delay and post-contrast enhancement, highlighted a hyperintense signal within the left cochlear region and the left posterior semicircular canal.

**Conclusion:**

The investigative results substantiate an infarction in the territory of the cochlear artery, precipitated by a vaso-occlusive event, thereby reinforcing the vascular hypothesis of cochleovestibular artery syndrome. This case underscores the congruence between clinical observations and delayed post-contrast MRI findings.

## Objective

To present a case of sudden sensorineural hearing loss (SNHL) where a delayed post-contrast magnetic resonance imaging (MRI) with high-resolution 3D fluid-attenuated inversion recovery (FLAIR), performed 4 h post-injection, morphologically substantiated the instrumental and audiological findings indicative of an infarction in the cochlear artery territory.

## Patient

This report details a 24-year-old male with sickle cell disease due to compound heterozygous SC, exhibiting 46% hemoglobin C and 8.9% G6PD-free fetal hemoglobin. His medical history is notable for a singular vaso-occlusive episode six years prior, which was complicated by medullary necrosis. Additionally, he has been diagnosed with sickle cell retinopathy and has an aneurysmal bone cyst of the humerus. This patient had only undergone a single transfusion episode and was neither followed up nor managed at our hospital for their sickle cell disease.

### Diagnostic

The patient presented at the emergency department with a 24-hour history of acute onset deafness and tinnitus of the left ear. There were no accompanying vestibular or neurological symptoms reported. Videonystagmography revealed right horizontal nystagmus, elicited by high-frequency skull-vibration (100 Hz). Halmagyi’s sign was negative. Otoscopy was normal. The tuning fork examination indicated sensorineural hearing loss in the left ear.

The patient had no other neurological signs, with a Glasgow Coma Scale score of 15, no headache, no decrease in visual acuity, no focal signs, no sensorimotor deficit, no peripheral facial paralysis, no cerebellar syndrome, and no pyramidal syndrome.

**Fig. 1:**
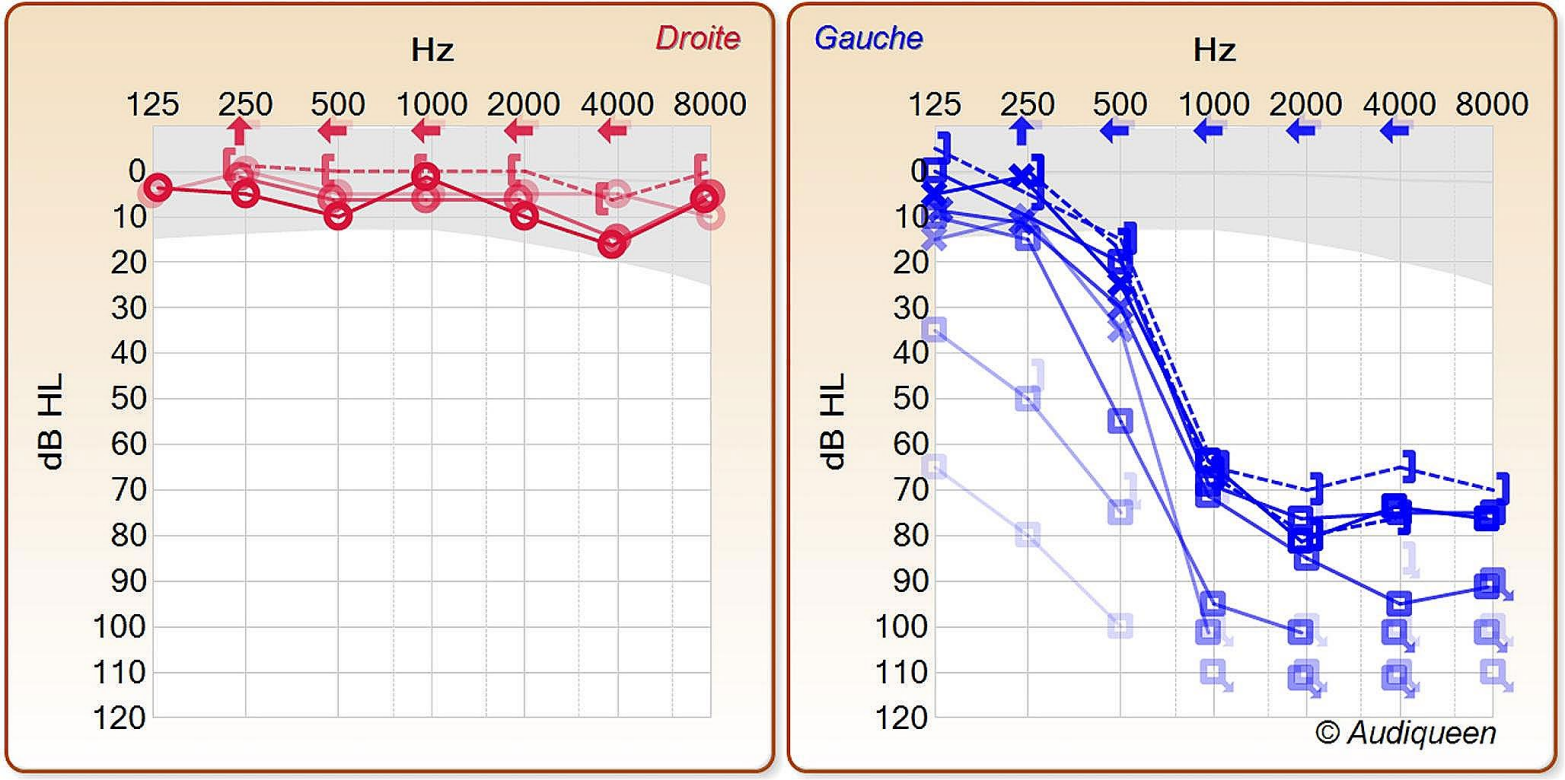
Pure tone audiometry initially revealed a profound sensorineural hearing loss (pale blue at diagnosis), which exhibited progressive recovery during assessments conducted in the subsequent days, weeks, and months (becoming progressively darker blue)

Pure tone audiometry (PTA) confirms a left SNHL (Fig. 1).

Laboratory investigations revealed a hemoglobin level of 13.1 g/dL, a platelet count of 497.10^9^/L (normal range: 150–400), and elevated lactate dehydrogenase (LDH) at 301 U/L (normal range: 135–225 U/L), with no electrolyte imbalances detected.

**Fig. 2:**
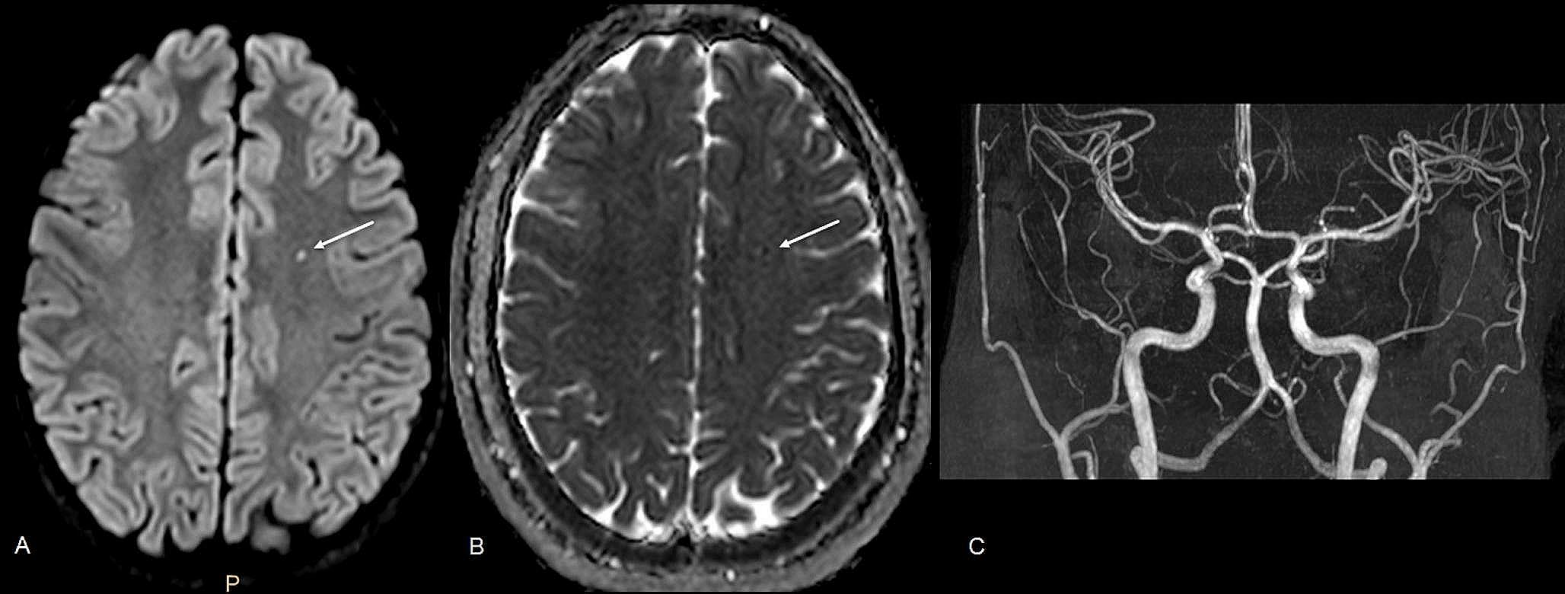
1.5 Tesla brain MRI with diffusion-weighted sequence displaying a punctiform lesion at b1000 (**A**), exhibiting restricted diffusion on ADC map (**B**), and no evidence of brain vessel occlusion on 3D Time Of Flight (TOF) imaging (**C**) Diagnosis: recent small ischemic stroke affecting the left frontal lobe

Due to the increased risk of arterial occlusion associated with sickle cell disease, cerebral MRI was conducted to rule out occlusive events within the territory of the anterior inferior cerebellar artery (AICA) or other potential ischemic lesions. The 1.5 Tesla MRI (Ingenia 1.5T MRI, Philips Healthcare, Best, The Netherlands) did not reveal ischemic lesions in the AICA territory; however, it did demonstrate a recent punctiform ischemic stroke in the left frontal area (Fig. 2). Vascular integrity within the cerebral vasculature and internal auditory canal was verified. There was no hypersignal of the labyrinth on the standard FLAIR sequences.

Following neurologic consultation, aspirin therapy at a dosage of 75 mg daily was initiated for the management of the ischemic stroke.

**Fig. 3:**
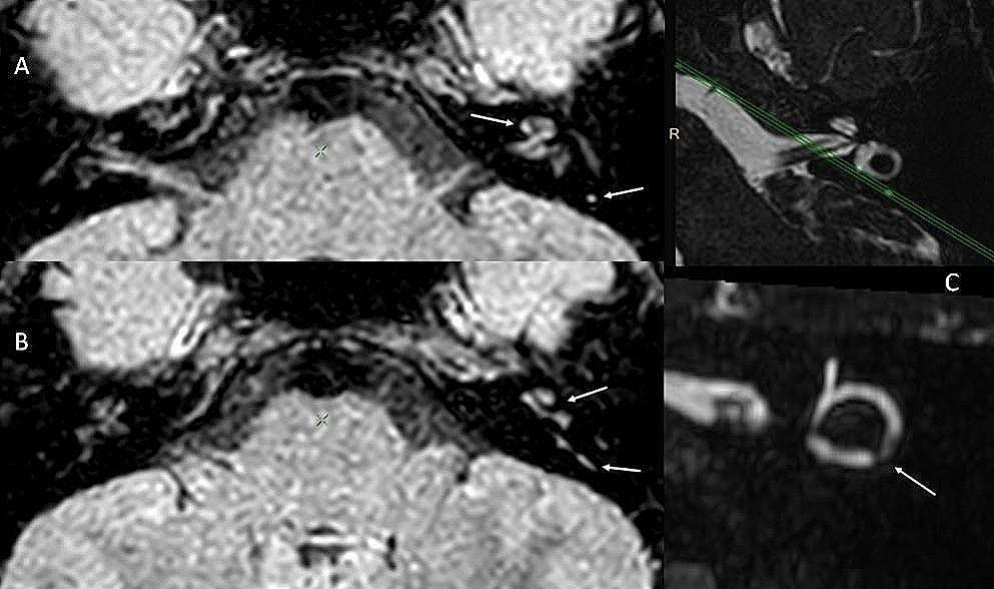
1.5 Tesla MRI performed 4 h post-intravenous gadolinium injection revealed hyperintensity within the left cochlea and posterior semicircular canal (arrows) on 3D FLAIR imaging (**A**) (**B**), and a minor fluid amputation from the left posterior semicircular canal (arrow) visualized on 3D DRIVE imaging reconstruction in the axis of the canal (**C**)

A 1.5 Tesla MRI of the ears was conducted 48 h post-symptom onset, with 3D High-Resolution (HR) DRIVE and 3D FLAIR HR sequences, 4 h after intravenous administration of gadolinium contrast (Dotarem 20 mL, Guerbet, Villepinte, France). The imaging revealed hyperintensity in the left cochlea and left posterior semicircular canal (SSC) on the 3D FLAIR sequence. Additionally, a subtle discontinuity in the fluid signal was observed at the inferior aspect of the posterior SSC arch on the 3D DRIVE sequence (Fig. 3).

**Fig. 4:**
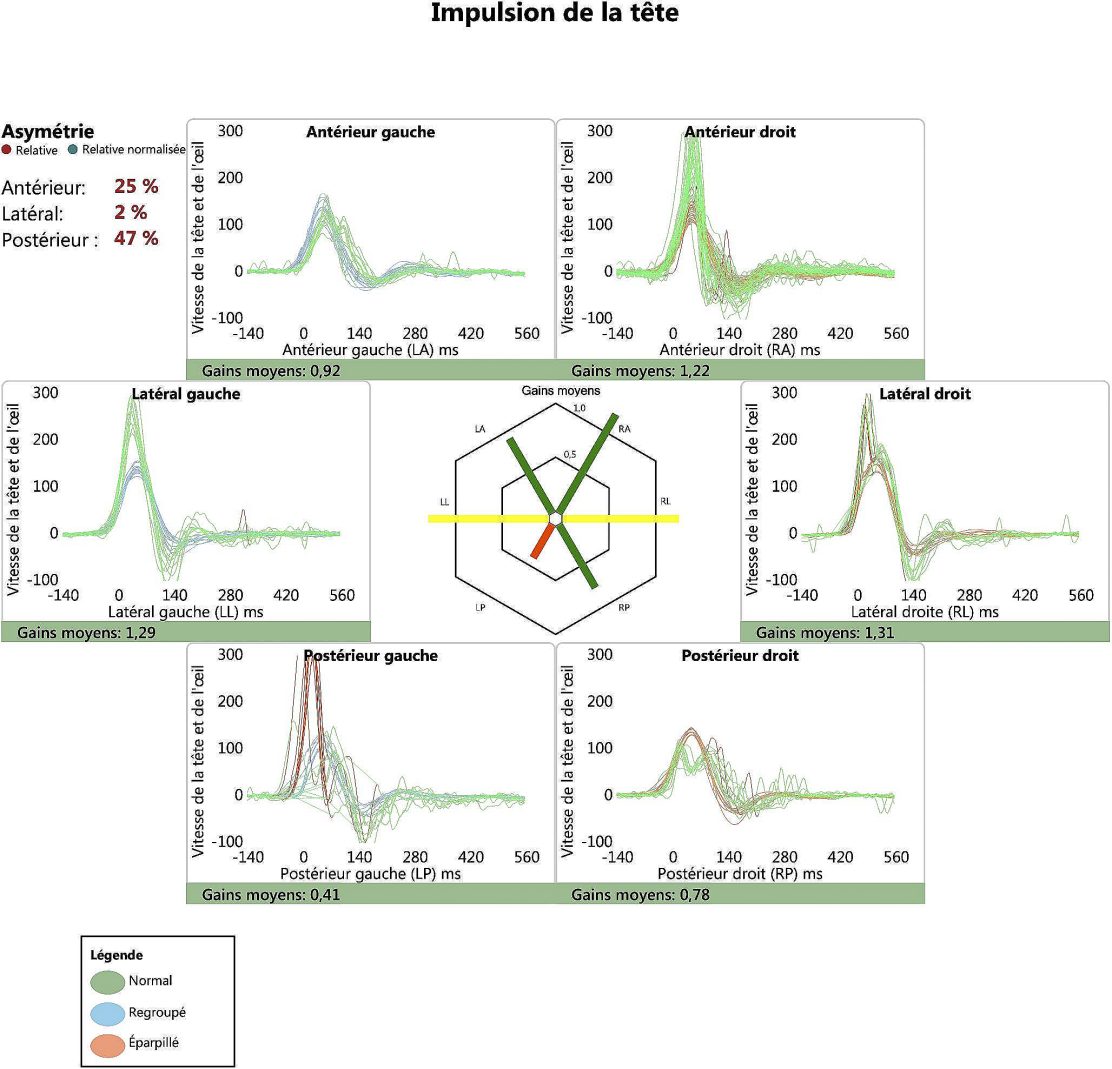
Video-Head-Impulse-Test showing an isolated deficit of the left posterior semicircular canal characterized by reduced gain and covert-saccades

A video-Head-Impulse-Test (vHIT) (ICS Impulse, Otometrics, Taastrup, Denmark) was performed, confirming the isolated functional impairment of the left posterior SCC (Fig. 4).

The diagnosis of sudden unilateral SNHL by acute occlusion of the cochlear artery was therefore confirmed instrumentally (PTA and vHIT) and morphologically (3T MRI).

## Intervention

Corticosteroid therapy was not initially proposed as it carries the risk of exacerbating the hyperviscosity syndrome and causing a severe vaso-occlusive crisis. Therapeutic phlebotomy was initiated in the emergency department, aiming to reduce hemoglobin levels to below 11.5 g/dL. This objective was achieved following the removal of 650 mL of blood (450 mL on the first day and 200 mL on the second day). The main objective of the phlebotomies was primarily to prevent further organ damage from this vaso-occlusive crisis. There were no adverse effects from these phlebotomies.

Discharge home was authorized on day 2 with auditory rest recommended, and a 10-day corticosteroid therapy (prednisolone 120 mg daily).

Following audiometric assessments on the 7th and the 11th days post-treatment initiation stable auditory thresholds were observed. A supplementary treatment of five trans-tympanic injections of Dexamethasone 4 mg/mL spaced 48 h was executed. Audiometric evaluation after injection at the first and the 7th month revealed normalization of low frequency and very slight improvement of high frequency (Fig. 1). vHIT at the 7th month showed no improvement compared to the initial one. A hearing aid was proposed approximately 7 months after the episode. However, the patient did not wish to procure it.

## Discussion

The relationship between sickle cell disease and hearing loss has been clearly documented (between 12 and 66% among individuals with sickle cell disease), and most of the cases have progressive bilateral hearing loss (progressive cochlear damage by hypoxia stemming from the sickle shape of the red cells) [1].

Sickle cell disease is also recognized as a contributing factor to sudden SNHL, with a reported incidence of 2.2% among pediatric cases [2, 3]. In cases of sudden bilateral sensorineural hearing loss, expeditious cochlear implantation is advocated in sickle cell patients, owing to the heightened risk of precipitous cochlear sclerosis [4]. In the general population, the etiologies of sudden SNHL are markedly heterogeneous and frequently elusive; idiopathic sudden sensorineural hearing loss is the predominant diagnosis [5–7]. In individuals with sickle cell disease, sudden SNHL is predominantly attributed to vaso-occlusive crises. As early as the 1970s, Berry reported the presence of sensorineural hearing loss in sickle cell patients, noting that hearing loss in the high frequencies may quite possibly be related to cochlear thrombosis in microcirculations [8].

Regarding the patient in question, it is substantiated that the individual experienced a vaso-occlusive crisis, which is further corroborated by the occurrence of a concomitant frontal cerebral infarction. The crisis likely involved the common cochlear artery, which supplies the cochlea and the posterior semicircular canal [7, 9].

This assertion is supported by clinical instrumental findings, including auditory impairment in PTA and isolated dysfunction of the posterior semicircular canal as assessed by vHIT, and by radiological (MRI) evidence indicating the involvement of the posterior semicircular canal and cochlea.

Furthermore, the utilization of delayed post-contrast MRI of the inner ear using 3D FLAIR HR sequences is being refined for the in vivo delineation of endolymphatic and perilymphatic fluids [10]. This diagnostic modality has demonstrated utility in the diagnosis of hydropic ear diseases [11, 12] and their auditory sequelae, albeit certain images may present interpretative challenges.

In emergency presentations of sudden SNHL with a strong suspicion of vascular etiology, this imaging technique permits the in vivo examination of inner ear fluids during the crisis. This could provide invaluable insights into the pathophysiological underpinnings of sudden SNHL and its etiological assessment.

In the present case, the clinical-radiological congruence is particularly evident, reinforcing the notion that delayed post-contrast MRI with 3D FLAIR HR sequence is capable of illustrationg the repercussions of localized arterial occlusion within the inner ear. Additionally, Eliezer et al. have documented an analogous presentation in a 60-year-old female patient with rheumatoid arthritis [13]. This case is differentiated by the hematologic pathology of the subject, bolstering the vascular etiology for what they have termed “Cochleovestibular artery syndrome”.

The absence of vertigo reported by the patient can be explained by the limited involvement of the posterior labyrinth (only the posterior semicircular canal), the lower gain of the posterior semicircular canal compared to the lateral canal, its non-alignment with the axis of head rotation, and the patient’s focus on his hearing loss which may have led to overlooking any added sensation of vertigo.

The right horizontal vibratory nystagmus indicates vestibular asymmetry affecting the left labyrinth. Despite no evidence of lateral canal involvement at high frequencies (vHIT), it may exist at very high frequencies (vibration-induced nystagmus). Cochlear ischemia also explain vestibular asymmetry by impairing the blood-labyrinth barrier and the stria vascularis, disrupting the endolymph (ionic, pressure, potential) balance, which affects all labyrinthine structures [14, 15].

## Conclusion

This clinical case elucidates several pivotal insights. Primarily, sudden SNHL, notably in an individual with sickle cell disease, mandates prompt cerebrovascular evaluation via brain MRI to rule out stroke during a vaso-occlusive crisis. Moreover, there is a discernible clinical-radiological congruity between the PTA-VHIT duo and the delayed post-contrast MRI of the inner ears utilizing a 3D FLAIR HR sequence in the acute arterial occlusion of the cochlear artery. This alignment furnishes novel avenues for the application of this imaging modality in the diagnostic process of sudden SNHL.
